# A Novel Graphene-Based Nanomaterial Modified Electrochemical Sensor for the Detection of Cardiac Troponin I

**DOI:** 10.3389/fchem.2021.680593

**Published:** 2021-05-14

**Authors:** Jing Li, Shenwei Zhang, Li Zhang, Yu Zhang, Hua Zhang, Chuanxi Zhang, Xuexi Xuan, Mingjie Wang, Jinying Zhang, Yiqiang Yuan

**Affiliations:** ^1^ Department of Cardiology, The Seventh People’s Hospital of Zhengzhou, Zhengzhou, China; ^2^ Department of Cardiology, The First Affiliated Hospital of Zhengzhou University, Zhengzhou, China; ^3^ Department of Cardiology, Chest Hospital of Henan Provincial, Zhengzhou, China

**Keywords:** aptamer electrochemical sensor, reduced graphene oxide, troponin I, mos2, glassy carbon electrode

## Abstract

Acute myocardial infarction has a high clinical mortality rate. The initial exclusion or diagnosis is important for the timely treatment of patients with acute myocardial infarction. As a marker, cardiac troponin I (cTnI) has a high specificity, high sensitivity to myocardial injury and a long diagnostic window. Therefore, its diagnostic value is better than previous markers of myocardial injury. In this work, we propose a novel aptamer electrochemical sensor. This sensor consists of silver nanoparticles/MoS_2_/reduced graphene oxide. The combination of these three materials can provide a synergistic effect for the stable immobilization of aptamer. Our proposed aptamer electrochemical sensor can detect cTnl with high sensitivity. After optimizing the parameters, the sensor can provide linear detection of cTnl in the range of 0.3 pg/ml to 0.2 ng/ml. In addition, the sensor is resistant to multiple interferents including urea, glucose, myoglobin, dopamine and hemoglobin.

## Introduction

Troponin is a regulatory heteropeptide protein found in myogenic fiber filaments that plays a pivotal role in the interaction between actin and myosin. It controls contraction and relaxation of skeletal and cardiac muscles. Troponin is a complex that includes three subunits: ion-binding troponin (cTnC), troponin that inhibits actin-myosin interaction (cTnI) and tropomyosin (cTnT), which is used to bind the myosin complex to promyosin and promote myocardial contraction (Fan et al., 2018; Miao et al., 2019; Phonklam et al., 2020). It has been demonstrated that cTnI is a specific biomarker for myocardial injury in dogs, cats, horses, pigs, goats, mice and cattle. Damage to myocardial cell integrity is followed by partial release of cTnI into the blood, and elevated cardiac troponin concentrations in peripheral blood indicate myocardial cell damage (Palladino et al., 2018; Karimi-Maleh et al., 2020a; Çimen et al., 2020). Since myocardial infarction is one of the most important factors leading to myocardial cell destruction, monitoring of cTnI blood concentrations is particularly important for the early detection of myocardial infarction (Ye et al., 2018; Lee et al., 2019; Sun et al., 2019a; Karimi-Maleh et al., 2020b, 2021). There is evidence that even small elevations in cTnI may be associated with poor prognosis. However, as with most other current assays, their most significant shortcoming lies within the first few hours of acute myocardial infarction onset. Current cTnI assays are not effective in detecting elevated blood concentrations and are not effective in monitoring changes in lower concentrations of cTnI. Therefore, the development and application of next-generation cTnI assays is imminent (Negahdary et al., 2017; Zhang et al., 2018; Fu et al., 2019b; Sun et al., 2019b; Zhou et al., 2020).

Radioimmunoassay is a method by which an isotope-labeled antigen is added to an unlabeled antigen to cause a competitive inhibition reaction with the antibody. The radioimmunoassay is equal to the advantages due to its good utility and specificity, as well as its high sensitivity. However, the method has disadvantages such as the existence of radioactive contamination and short half-life of isotopes, which to a certain extent limit the development and application of radioimmunoassay (Qiao et al., 2018; Sarangadharan et al., 2018; Fu et al., 2019a; Wang et al., 2019; Mokhtari et al., 2020; Xu et al., 2020). Fluorescent immunoassay is mainly used for the diagnosis of infectious diseases due to its high sensitivity, and it is one of the oldest labeled immunoassays. However, the detection limit of fluorescence immunoassay is not low enough. Moreover, it is difficult to store fluorescent samples for a long time. Electrochemical immunosensor is an application that combines electrochemiluminescence measurement with immunosensor. It combines the advantages of high sensitivity of electrochemiluminescence and high selectivity of immunoassay, which has attracted the attention of many researchers (Dhawan et al., 2018; Regan et al., 2018; Karimi-Maleh et al., 2020c; Wang et al., 2020). The unique physicochemical properties of nanomaterials make it a promising application in the development of high-performance electrochemical and electrochemiluminescent sensors.

Two-dimensional nano has been widely studied for its high specific surface area and compatibility with miniaturized devices (Khodadadi et al., 2019; Tahernejad-Javazmi et al., 2019). Among them, MoS_2_, in which Mo atomic layers are arranged in a hexagonal shape between S atomic layers, has an ultrathin planar structure. It is sensitive to the surrounding environment and becomes a suitable base material for building aptamer sensors (Cai et al., 2018; Chekin et al., 2018). However, MoS_2_ has low electrical conductivity and Van Der Waals between the layers tend to agglomerate it. Reduced graphene (rGO) with active edge sites and excellent electrical properties can effectively improve the electrochemical activity of MoS_2_. The composite of other nanomaterials in rGO can reduce the layer-layer interaction force, so the performance of rGO can be further ensured (Fathil et al., 2017; Lopa et al., 2019). Among them, silver nanomaterial materials are often used in the design of electrochemical sensors due to their cheap price and good electrocatalytic properties (Zhou et al., 2018; Yan et al., 2019).

This work synthesized AgNPs/MoS_2_/rGO nanocomposites. By combining the excellent properties of the composite (large surface area and good electrical conductivity) with the aptamer (high affinity and specificity), a label-free electrochemical aptamer sensor was developed for the sensitive and selective detection of cTnI.

## Materials and Methods

cTnI and DNA oligonucleotides were obtained from Yeyuan Biotech. Urea, thrombin, myoglobin, horseradish peroxidase, l-cysteine, hemoglobin, and prostate-specific antigen were ordered from Alading Co. Ltd. Graphene oxide (GO) powder was purchased from Xianfeng Nano Tech Co. Ltd. All other reagents are analytically pure and can be used without additional purification. 0.1 M Tris-HCl buffer solution has been used for dissolving amino-modified cTnI aptamer (AcTnl).

Sequence of the AcTnl is: 5′-NH_2_-C_6_H_12_-CGTGCAGTACGCCAACCTTTCTCATGCGCGCTGCCCCTCTTA-3′.

All electrochemical tests, including cyclic voltammetry (CV), differential pulse voltammetry (DPV), and electrochemical impedance spectroscopy (EIS) were performed using the CHI660 electrochemical analyzer/workstation with three-electrode system. SEM image has been recorded using a ZEISS MERLIN.

Synthesis of AgNPs/MoS_2_/rGO nanocomposite: 50 mg of AgNO_3_ was dissolved in 20 ml of ethanol and 100 mg of octadecylamine was added with vigorous stirring. After complete dissolution, 50 mg of glucose and 60 mg of glycine were added and sonicated. The solution was transferred to a hydrothermal kettle and heated at 120°C for overnight. Silver nanoparticles (AgNPs) were obtained after filtration. MoS_2_/rGO was prepared by adding 10 mg Na_2_MoO_4_.2H_2_O and 20 mg l-cysteine into 20 ml of GO dispersion (0.5 mg/ml, DMF). The mixture was then transferred into a hydrothermal kettle and heated at 100°C for 5 h. The MoS_2_/rGO was collected after the filtration. The AgNPs/MoS_2_/rGO nanocomposite was prepared by adding AgNPs into MoS_2_/rGO composite dispersion after 1 h sonication.

Electrode fabrication: A glassy carbon electrode (GCE) has been used for sensor fabrication. Specifically, a certain amount of AgNPs/MoS_2_/rGO nanocomposite was drop casted on a GCE and dried naturally. Then, a certain amount of AcTnl was drop casted on the AgNPs/MoS_2_/rGO/GCE. After drying, the AcTnl/AgNPs/MoS_2_/rGO was incubated in the cTnl solution with different concentration for 1 h before electrochemical signal recording.

## Results and Discussion

**FIGURE 1:**
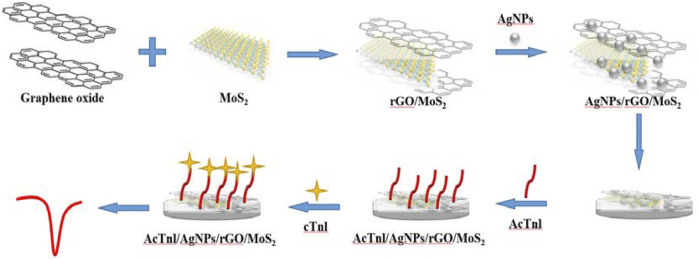
Schematic diagram for the preparation of the AcTnl/AgNPs/MoS_2_/rGO sensor for cTnI detection.

Figure 1 demonstrates the preparation and sensing strategy of this label-free electrochemical aptamer sensor. AgNPs/MoS_2_/rGO immobilized with GCE can immobilize the AcTnl aptamer on the electrode surface via Ag-N bond. In this process, the electrochemical signal of [Fe(CN)_6_]^3-/4-^ decreases because the electron transfer is hindered by the nucleobases. When AcTnl/AgNPs/MoS_2_/rGO is immersed in the cTnI solution, the electrochemical signal is further reduced due to the formation of AcTnI-cTnI complexes on the electrode surface that further hinder electron transfer. By calculating the relationship between peak current and cTnI concentration, the detection of cTnI concentration can be achieved.

**FIGURE 2:**
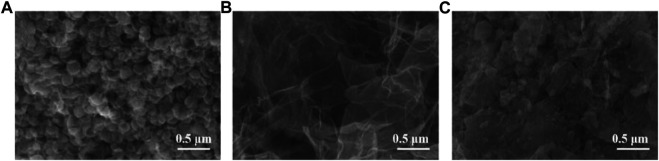
SEM images of **(A)** AgNPs **(B)** MoS_2_/rGO, and **(C)** AgNPs/MoS_2_/rGO.

Figure 2 shows the SEM images of AgNPs, MoS_2_/rGO, and AgNPs/MoS_2_/rGO. The structure of silver nanoflowers can be clearly observed in Figure 2A. The SEM images of MoS_2_/rGO show the typical wrinkled and folded structure of graphene and the layer-like structure of MoS_2_/rGO. The tight contact between MoS_2_ and rGO is favorable for electron transfer. The SEM images of AgNPs/MoS_2_/rGO nanocomposites clearly observe that AgNPs are wrapped by MoS_2_/rGO composites.

**FIGURE 3:**
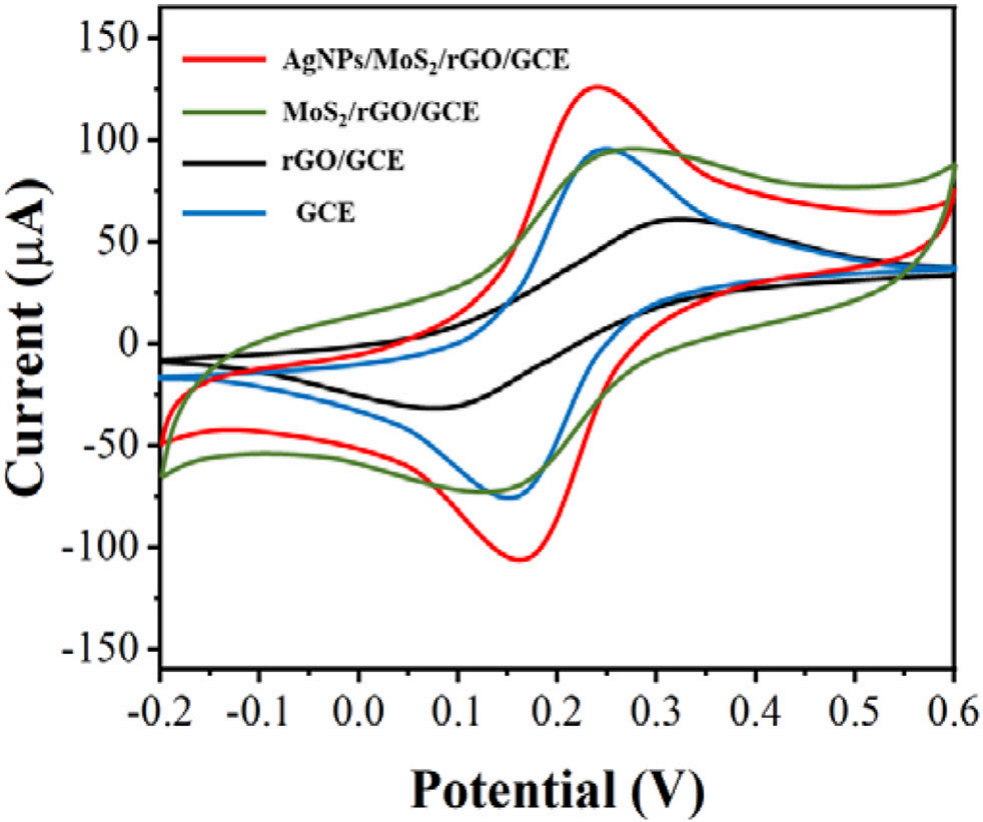
CV of GCE, rGO/GCE, MoS_2_/rGO/GCE and AgNPs/MoS_2_/rGO/GCE in 5.0 mM [Fe(CN)_6_]^3-/4-^. Scan rate: 50 mV/s.

We tested the electrochemical behavior of the electrode surface during sensor assembly using a 5.0 mM [Fe(CN)_6_]^3-/4-^ solution containing 0.1 M KCl (Figure 3). The redox peak current of the modified GCE was significantly increased due to the excellent conductivity and electron transfer of rGO. After the modification of GCE with MoS_2_/rGO and AgNPs/MoS_2_/rGO, the currents increased significantly in turn, which was caused by the large specific surface area of MoS_2_ and the excellent electrochemical properties of AgNPs.

**FIGURE 4:**
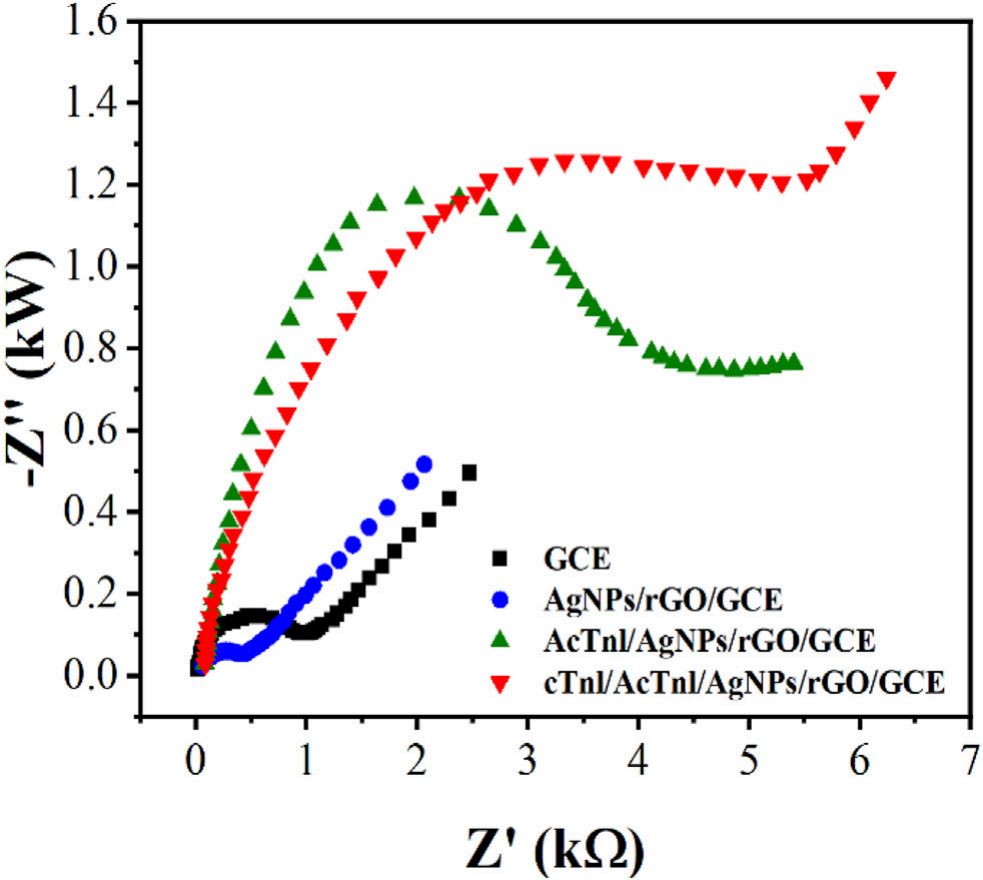
EIS of GCE, rGO/GCE, MoS_2_/rGO/GCE and AgNps/MoS_2_/rGO/GCE in 5.0 mM [Fe(CN)_6_]^3-/4-^.

The electrochemical properties of the sensor was further investigated by EIS. In EIS, semicircular and straight regions indicate electron transfer-limited processes and mass transfer control processes (Demirbakan and Kemal Sezgintürk, 2020; Lee et al., 2020), respectively. Considering the variation of the electron transfer resistance (R_ct_), the EIS method can provide important information to demonstrate the interaction between the aptamer and the target protein. As shown in Figure 4, bare GCE presents a small semicircle with an R_ct_ value of 266 *Ω*. Due to the excellent properties and synergistic effects of rGO, MoS_2_ and AgNPs, the R_ct_ value decreases to 91 *Ω* after AgNPs/MoS_2_/rGO modification, indicating that the electron transfer effect is enhanced. This is consistent with the CV results of GCE and AgNPs/MoS_2_/rGO/GCE. While the presence of AcTnI leads to an increase in the spatial site blocking effect and hinders electron transfer, the R_ct_ increases sharply to occur at 3.31 kΩ, indicating the binding of AcTnI and AgNWs on the electrode surface. With the binding of the target protein on the AcTnI/AgNPs/MoS_2_/rGO/GCE surface, a continued increase in R_ct_ to 5.26 kΩ can be observed, indicating a successful binding with the target protein to its aptamer, which hinders the electron transfer.

**FIGURE 5:**
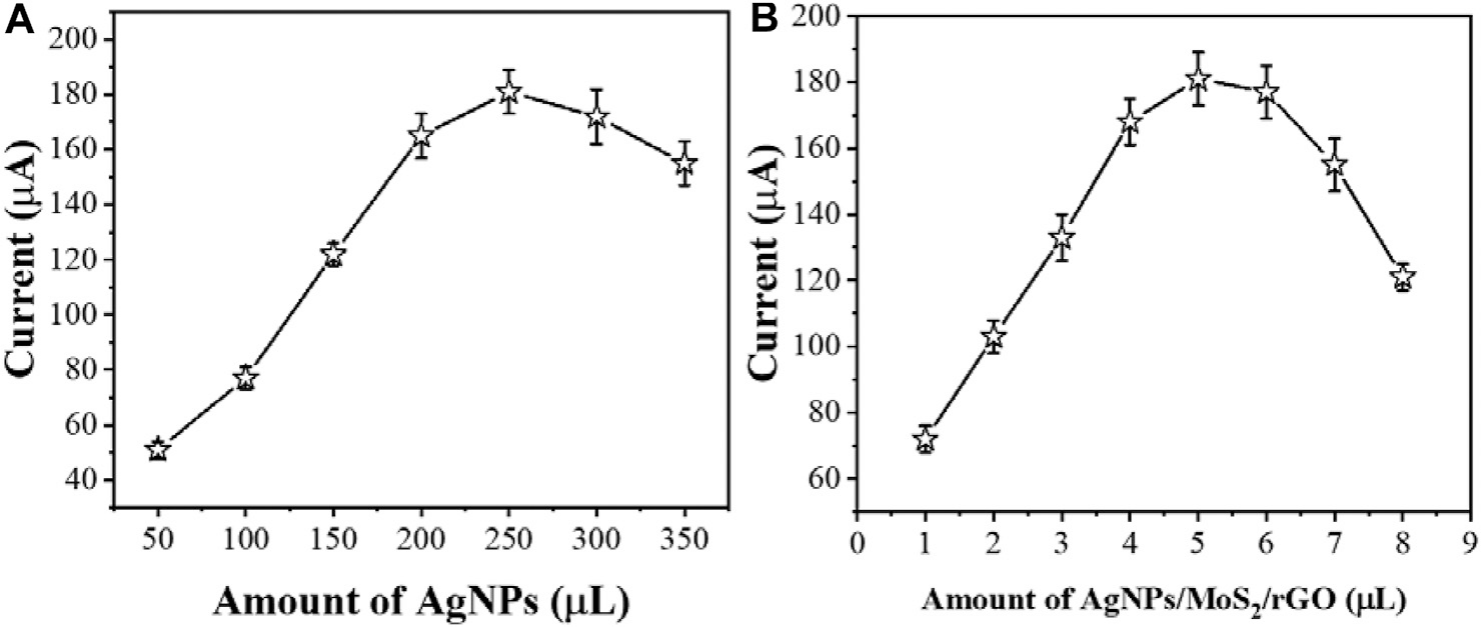
The effect of **(A)** amount of AgNPs and **(B)** AgNPs/MoS_2_/rGO composite for sensing performance (*n* ≥ 3).

In order to improve the sensitivity of detecting cTnI, different parameters were optimized. Figure 5A shows the aptamer electrodes assembled with different concentrations of AgNPs in the MoS_2_/rGO dispersion. Although AgNPs have excellent electrochemical properties, when AgNPs exceed a certain concentration it may cause a high stacking density, resulting in a lower detection efficiency of the sensor. Therefore, we chose to add 250 μLof AgNPs into the MoS_2_/rGO dispersion.

The amount of modification of AgNPs/MoS_2_/rGO is an important factor affecting the immobilization of AcTnI. Figure 5B shows the electrochemical response of the proposed aptamer sensor AgNPs/MoS_2_/rGO at different modification amounts. As the concentration of AgNPs/MoS_2_/rGO increases, the electron transfer resistance of the interface may increase, while there may be some interference with the detection of cTnI. Therefore, 5 μL of AgNPs/MoS_2_/rGO dispersion was obtained as the optimal condition.

**FIGURE 6:**
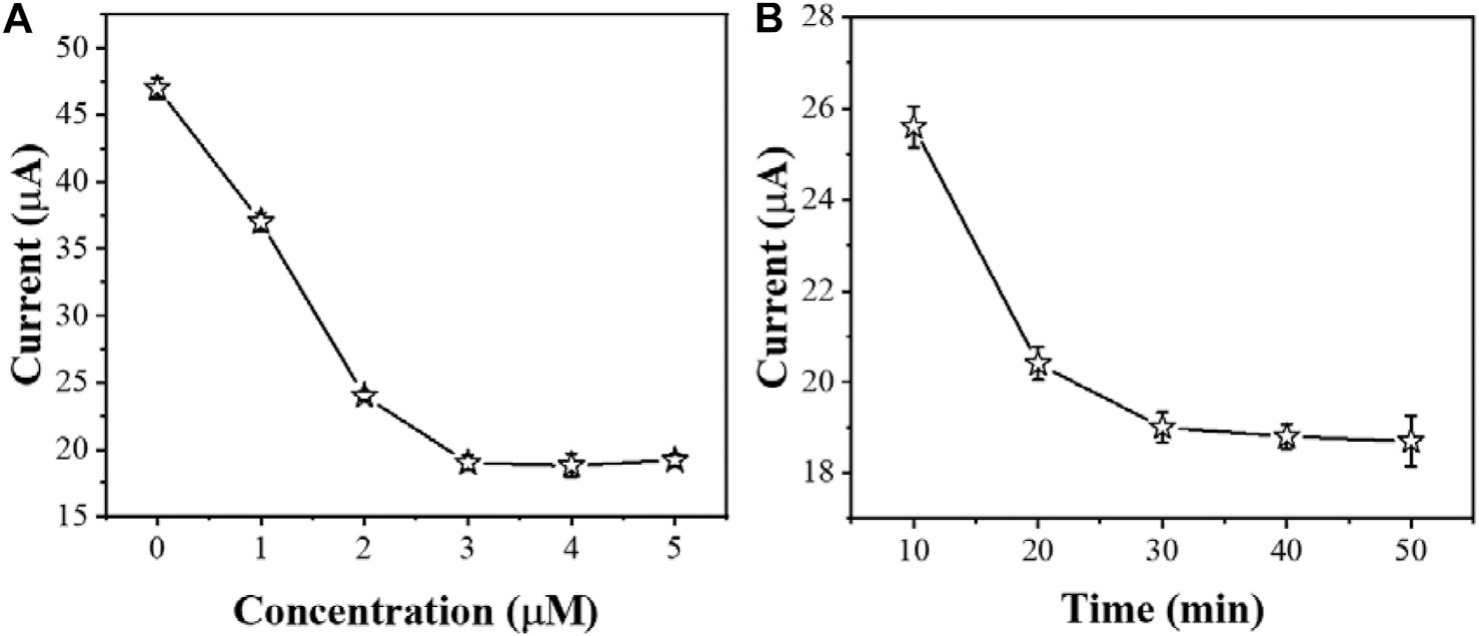
The effect of **(A)** amount of AcTnI and **(B)** incubation time for sensing performance (*n* ≥ 3).

The concentration of AcTnI affects the charge transfer efficiency of the electrode interface for detecting cTnI. Figure 6A shows the effect of different AcTnI concentrations on the DPV signal of AgNPs/MoS_2_/rGO. When the concentration reaches 3 μM, the DPV response is significantly reduced, indicating that the surface active site of the sensor may be fully occupied. Considering the size of the aptamer itself, the tightly packed surface prevents the aptamer from specifically binding cTnI. because we chose 3 μM AcTnI as the optimal concentration for the aptamer sensor.

The incubation time of cTnI is also another important factor affecting cTnI detection. As shown in Figure 6B, the electrochemical signal did not change significantly after the incubation time of AcTnI reached 30 min. This indicates that the bioaffinity between AcTnI on the electrode surface and the cTnI target saturated. On the other hand, the current response stabilizes due to the spatial potential resistance effect, leading to a decrease in sensitivity to the target. Therefore, we chose 30 min as the optimal incubation time.

**FIGURE 7:**
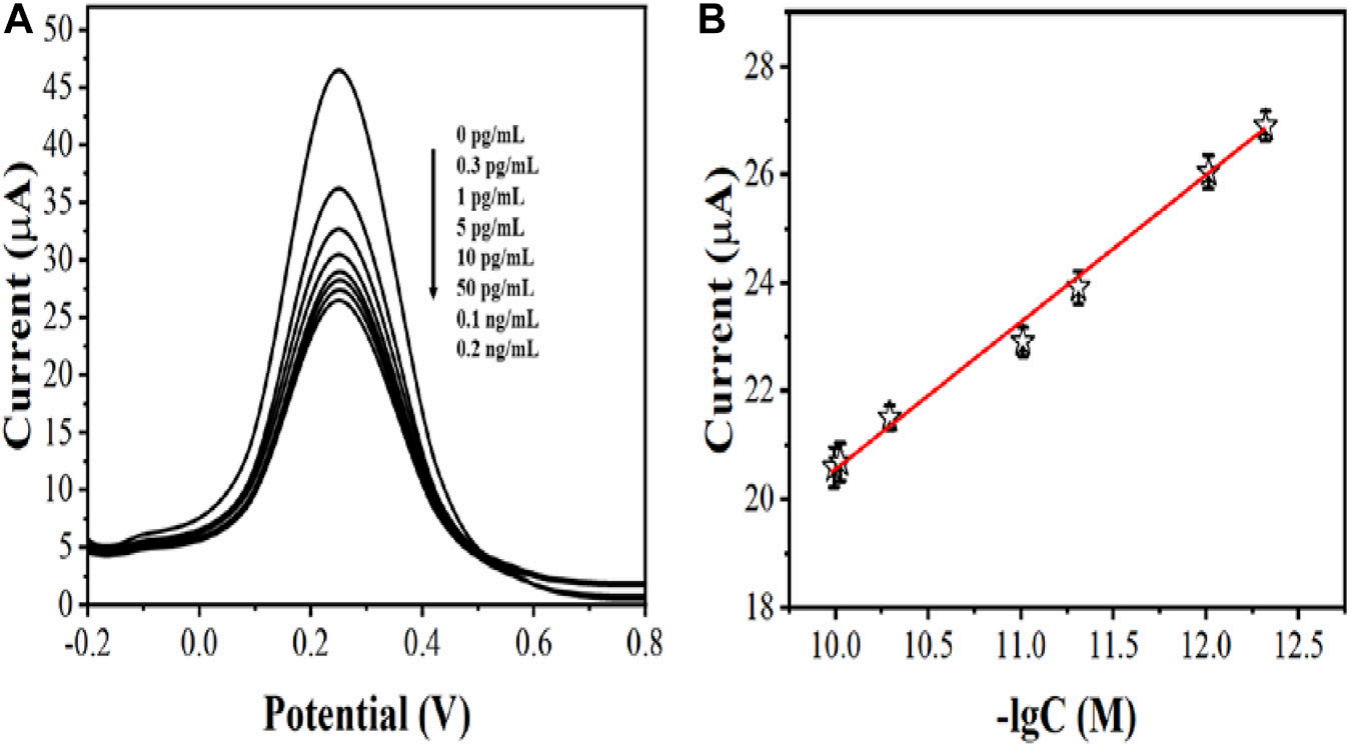
**(A)** DPV curves of AcTnI/AgNPs/MoS_2_/rGO/GCE toward 0 pg/ml, 0.3 pg/ml, 1 pg/ml, 5 pg/ml, 10 pg/ml, 50 pg/ml, 0.1 ng/ml, and 0.2 ng/ml **(B)** Linear relationship between current and logarithm of the cTnl concentrations (*n* ≥ 3).

After optimizing the parameters, we immersed AcTnI/AgNPs/MoS_2_/rGO/GCE in different concentrations of cTnI solutions. Then, we detected the electrochemical signal by DPV in a 5 mM [Fe(CN)_6_]^3-/4-^ solution containing 0.5 M KCl (Figure 7A). We recorded the linear relationship between the peak value of DPV and the negative logarithm of the concentration of the cTnI standard solution to establish a standard working curve (Figure 7B). It can be seen that the DPV signal correlates well with the negative logarithm of the cTnI concentration in the range of 0.3 pg/mL–0.2 ng/ml with the linear equation: Ip (μA) = −2.73 logc (g/ml) −6.41 (*R* ^2^ = 0.9947) and the limit of detection limit was 0.27 pg/ml.

To examine the performance of the aptamer sensor, we tested the reproducibility, stability and specificity of AcTnI/AgNPs/MoS_2_/rGO/GCE. The RSD of 1.72% was recorded by checking 5 parallel measurements on the same sensor, indicating that the electrochemical aptamer sensor for cTnI detection has good reproducibility. The stability of the prepared aptamer sensor was further evaluated by measuring the DPV current of the modified electrode after one month. The electrochemical signal before and after storage changed slightly with a 5.2% decrease in the peak DPV, demonstrating the good stability of the proposed electrochemical aptamer sensor for cTnI detection.

**FIGURE 8:**
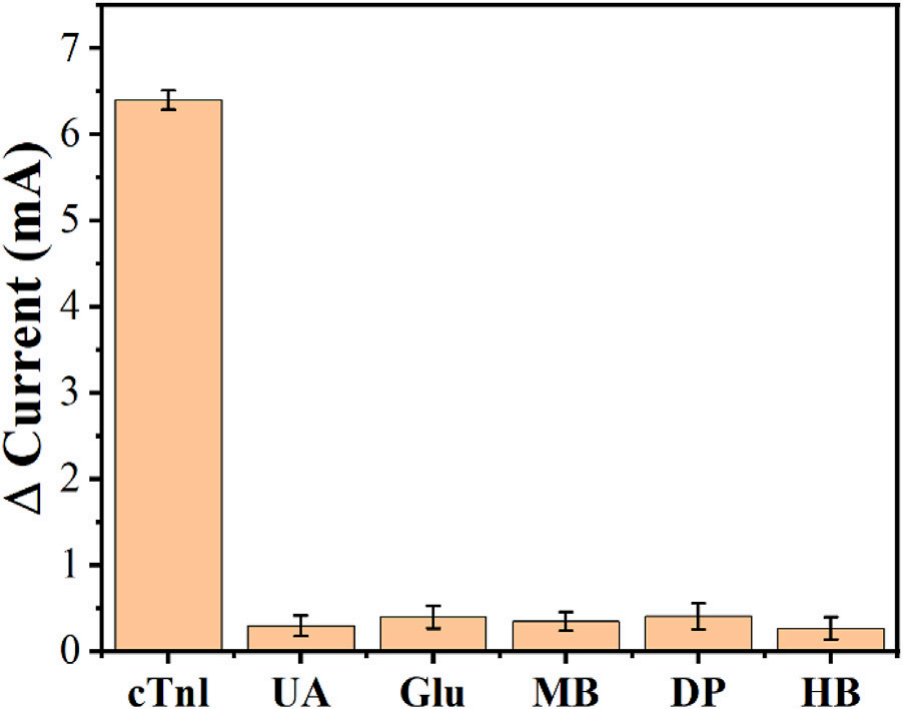
Anti-interference performance of the AcTnI/AgNPs/MoS_2_/rGO/GCE toward UA, Glu, MB, DP, and HB.

To determine the selectivity of the developed aptamer sensor, a series of proteins were used for comparison, such as: urea (UA), glucose (Glu), myoglobin (MB), dopamine (DP), and hemoglobin (HB). Among them, the concentration of cTnI was 50 pg/ml and the concentration of other infectants was 0.2 ng/ml due to the excellent specific discrimination between cTnI and AcTnI, the aptamer sensor for interferers showed negligible change in current response compared to cTnI, and the results are shown in Figure 8. The proposed aptamer sensor has good selectivity and specific anti-interference ability.

## Conclusion

In summary, an aptamer electrochemical sensor was constructed using AgNPs/MoS_2_/rGO nanocomposite. By combining the excellent properties of the composite (large surface area and good electrical conductivity) with the aptamer (high affinity and specificity), a label-free electrochemical aptamer sensor was developed for the sensitive and selective detection of cTnI. After the optimizations, the proposed aptamer sensor can linear detect cTnl between 0.3 pg/mL–0.2 ng/ml with a low limit of detection of 0.27 pg/ml. In addition, the proposed aptamer sensor has good selectivity and specific anti-interference ability.

## Data Availability

The original contributions presented in the study are included in the article/Supplementary Material, further inquiries can be directed to the corresponding authors.
